# Efficacy and safety of flow diverters in small intracranial aneurysms: a systematic review and single-arm meta-analysis

**DOI:** 10.3389/fneur.2025.1706462

**Published:** 2025-11-26

**Authors:** Kuerban Maimaitiaili, Maimaitiaili Nuermaimaiti, Adili Rouzi, Aisan Seyiti, Mairidan Yasheng, Xiong Guo, Moming Abulaiti, Ying Xiao, Hongbo Pan, Ainipai Sidike, Yanbing Hu, Dawutijiang Yilamu

**Affiliations:** 1 Department of Neurosurgery, The Second People’s Hospital of Kashgar Region, Kashgar, Xinjiang, China; 2 Department of Neurology, The Second People’s Hospital of Kashgar Region, Kashgar, Xinjiang, China; 3 Kashgar No. 8 Middle School, Kashgar, Xinjiang, China

**Keywords:** flow diverters, small intracranial aneurysms, intracranial aneurysms, FDs, meta-analysis

## Abstract

**Objective:**

This study aimed to evaluate the efficacy and safety of flow diverters (FDs) in the treatment of small intracranial aneurysms (≤10 mm).

**Methods:**

PubMed, Embase, Web of Science, and Cochrane Library were comprehensively searched up to July 2025. Eligible studies included retrospective cohort studies reporting angiographic and clinical outcomes of FD treatment in small intracranial aneurysms. Data analysis was conducted using STATA 15.0. Pooled proportions and 95% confidence intervals (CIs) were calculated using Freeman–Tukey double arcsine transformation. Publication bias was evaluated using funnel plots and Egger’s test.

**Results:**

Ten retrospective studies comprising 2,275 patients with 1,938 aneurysms were included. The pooled complete or near-complete occlusion rate was 86% (95% CI: 80–92%). The treatment-related mortality rate was 1% (95% CI: 0–2%), and the hemorrhagic event rate was 1% (95% CI: 1–2%). The ischemic event rate was 2% (95% CI: 1–3%), and the stroke rate was 3% (95% CI: 1–5%). The overall complication rate was 9% (95% CI: 5–12%), while 98% (95% CI: 94–100%) of patients achieved favorable functional outcomes. Egger’s test showed no significant publication bias (*p* = 0.791).

**Conclusion:**

FDs appear to be both effective and safe for the treatment of small intracranial aneurysms, achieving high occlusion rates and favorable functional outcomes with low rates of mortality and complications. However, given the high heterogeneity and retrospective nature of the included studies, further large-scale prospective studies are warranted to confirm these findings and refine treatment strategies.

## 1 Introduction

Intracranial aneurysms are among the most common cerebrovascular disorders, with a prevalence of approximately 3.2% in the adult population (1). Rupture can lead to aneurysmal subarachnoid hemorrhage (aSAH), which carries a high case fatality rate, the 30-day in-hospital mortality is about 20%, and prehospital deaths further increase the overall burden (2). With advances in neuroimaging, an increasing number of SIAs (≤10 mm in diameter) have been detected, accounting for 70–80% of unruptured aneurysms (3). Although their rupture risk is generally lower than that of large or giant aneurysms, rupture of small intracranial aneurysms (SIAs) can result in devastating consequences. The Unruptured Cerebral Aneurysm Study of Japan (UCAS Japan) reported an annual rupture rate of 0.36% for aneurysms <7 mm, yet emphasized the poor prognosis once rupture occurs, underscoring the clinical challenges in managing SIAs (4).

Current treatment options include microsurgical clipping and endovascular coiling. Microsurgical clipping achieves high occlusion rates but requires craniotomy, with greater risks in anatomically complex cases (5). Endovascular coiling offers a minimally invasive alternative, particularly wide-necked or morphologically complex aneurysms remain prone to incomplete occlusion and recurrence (6, 7).

Flow diverters (FDs) represent an alternative therapeutic strategy by reconstructing parent vessel hemodynamics, promoting intra-aneurysmal thrombosis, and facilitating vessel wall remodeling, leading to progressive occlusion. Their efficacy and feasibility in large and giant aneurysms are well established (8, 9). More recently, evidence has accumulated in SIAs. The prospective multicenter PREMIER trial (≤12 mm, predominantly small-to-medium aneurysms) demonstrated high complete occlusion rates and low permanent neurological morbidity, findings later supported by other series (10–12). However, systematic evaluations of long-term outcomes and complication profiles in SIAs remain limited.

Therefore, this study conducted a systematic review and single-arm meta-analysis to comprehensively evaluate the efficacy and safety of FD treatment in SIAs, aiming to provide evidence-based insights for clinical practice and future research.

## 2 Methods

This systematic review and meta-analysis was conducted in accordance with the Preferred Reporting Items for Systematic Reviews and Meta-Analyses (PRISMA) guidelines (13) and was prospectively registered in the PROSPERO database (registration number: CRD420251136570).

### 2.1 Search strategy

PubMed, Web of Science, EMBASE, and the Cochrane Library were searched from inception to July 15, 2025. Search terms combined MeSH and free-text words: “Intracranial Aneurysm” OR “Small intracranial aneurysms” AND “Flow diverter” OR “Pipeline Embolization Device.” The full search strategy is provided in Supplementary Table S1.

### 2.2 Eligibility criteria

Inclusion criteria: (1) adult patients with SIAs (≤10 mm); (2) treatment with an FD (e.g., Pipeline Embolization Device (PED), Pipeline Flex, FRED X); (3) reporting at least one of the following outcomes: complete occlusion (Raymond–Roy Grade I), incomplete/partial occlusion, perioperative complications (ischemia, hemorrhage, thrombosis), mortality, recurrence, or functional outcome (modified Rankin Scale, mRS); (4) ≥ 20 patients with SIAs; and (5) prospective or retrospective observational studies.

Exclusion criteria: large or giant aneurysms (>10 mm); non-intracranial aneurysms; non-FD interventions; insufficient data; case reports, reviews, meta-analyses, editorials, conference abstracts; and duplicate or overlapping cohorts.

### 2.3 Study selection and quality assessment

All records were imported into EndNote 21. Duplicates were removed, and two reviewers independently screened titles/abstracts and full texts, with disagreements resolved by a third reviewer. Quality assessment was performed using the Joanna Briggs Institute (JBI) Critical Appraisal Checklist for Case Series (14).

### 2.4 Data extraction

Two reviewers independently extracted data using a standardized electronic form, including study characteristics (author, year, country), patient demographics, aneurysm features, treatment details.

### 2.5 Statistical analysis

Analyses were performed using Stata 15.0. Outcomes were expressed as proportions, and pooled using single-arm meta-analysis. To stabilize variance, the Freeman–Tukey double arcsine transformation was applied. Pooled estimates with 95% CIs were reported. Heterogeneity was assessed with Cochran’s Q test and the I^2^ statistic; random-effects models (DerSimonian–Laird) were applied when I^2^ > 50%, otherwise fixed-effects models were used. Publication bias was assessed with funnel plots and Egger’s test.

## 3 Results

### 3.1 Study selection

**Figure 1:**
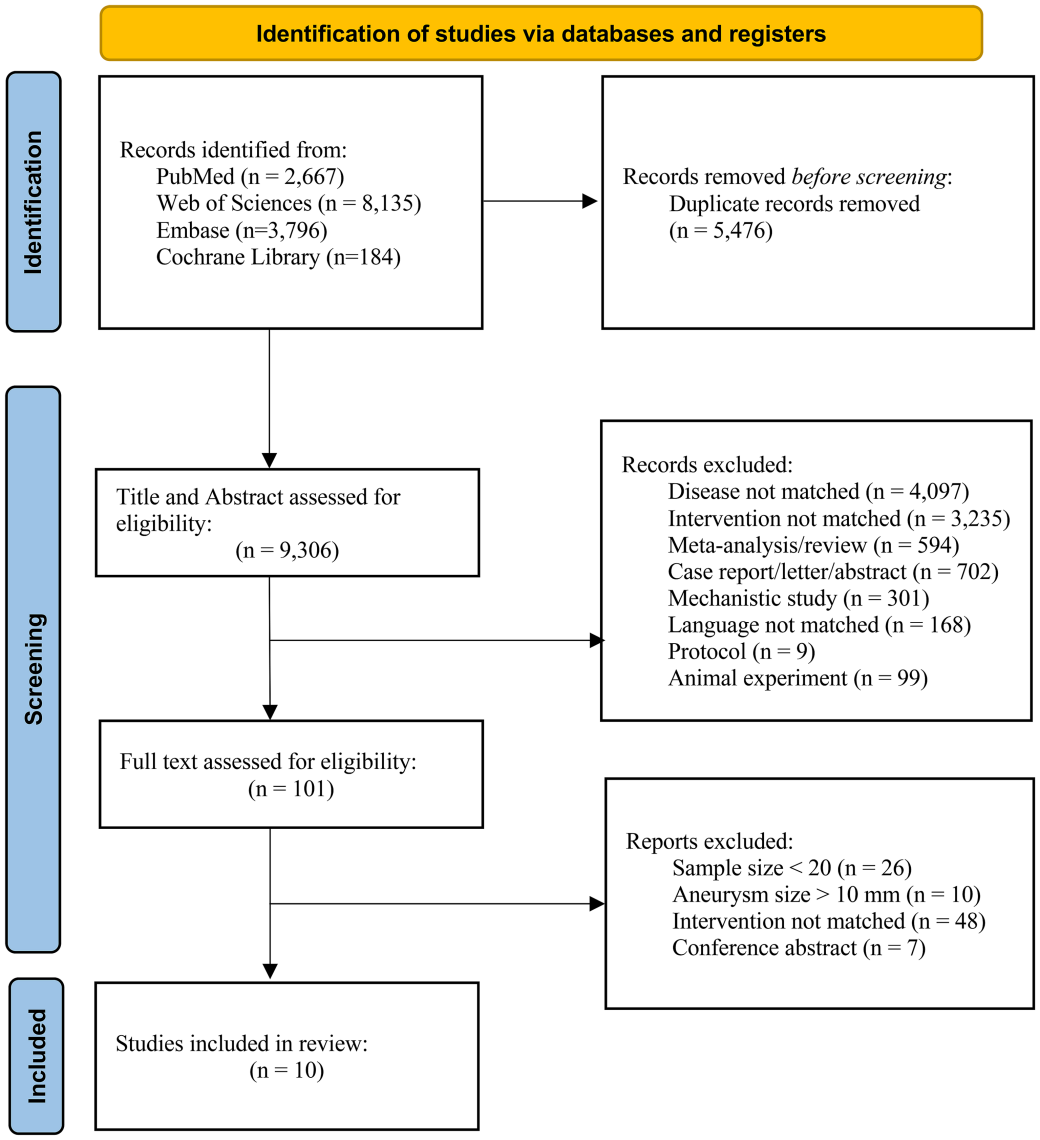
PRISMA flow diagram of study selection.

From 14,782 records, 5,476 duplicates were removed, leaving 9,306 for screening. After excluding 9,205 based on titles/abstracts, 101 full texts were assessed, and 10 studies met inclusion criteria (shown in Figure 1).

### 3.2 Study characteristics

The 10 retrospective studies (3, 15–23) included 2,275 patients with 1,938 SIAs. Five studies used PED, two FRED X, one PED Flex, and two reported mixed FD devices (SILK, Derivo, Surpass). Studies were conducted in the United States, Turkey, China, and Brazil. Study characteristics are summarized in Table 1.

**Table 1:** Basic characteristics information of included studies.

Study	*N*	*n*	Gender F/M	Size (mm)	Age (years)	Follow-up (m)	Rupture status	Circulation	DAPT	Flow-diverting stents	Country
Akgul et al. (15)	43	66	14/29	≤5	50.2 ± 12	26 (6–52)	Unrauptured + Ruptured	anterior circulation	Aspirin + Clopidogrel	SILK; Derivo; Pipeline; Surpass; FRED	Turkey
Brasiliense et al. (16)	205	121	165/40	≤7	55.7 ± 14.4	1	Unruptured Aneurysms	anterior + posterior circulation	NR	PED Flex	USA
Chalouhi et al. (17)	100	100	89/11	5.2 ± 1.5	17–80	6.3	Unruptured + Ruptured	anterior + posterior circulation	Aspirin + Clopidogrel	PED	USA
Clausen et al. (18)	77	53	56/21	≤10	58.9 ± 17.75	6	Unruptured + Ruptured	anterior + posterior circulation	Aspirin + Ticagrelor	FRED X	USA
Griessenauer et al. (19)	117	149	100/17	≤7	54 ± 14.5	6	Unruptured + Ruptured	anterior + posterior circulation	Aspirin + Clopidogrel	PED	USA
Kallmes et al. (20)	793	349	NR	5.2 ± 2.2	NR	12	Unruptured + Ruptured	anterior + posterior circulation	NR	PED	USA
Lin et al. (21)	41	53	38/3	5.34 ± 0.3	54.9 ± 1.6	4.0 ± 1.9	Unruptured + Aneurysms	anterior circulation	Aspirin + Clopidogrel	PED	USA
Roy et al. (22)	154	162	126/28	5.9 ± 4.0	56.8 ± 12.84	12	Unruptured + Ruptured	anterior + posterior circulation	Aspirin + Clopidogrel	FRED X	USA
Trivelato et al. (23)	93	131	86/7	8.04 ± 0.53	52.50 ± 1.32	12	Unruptured Aneurysms	Anterior + Posterior circulation	Aspirin + Clopidogrel	PED	Brazil
Zhang et al. (3)	652	754	448/204	6.78 ± 2.67	53.9 ± 10.3	8.26 ± 5.91	Unruptured + Ruptured	Anterior + Posterior circulation	aspirin + clopidogrel	PED Classic or PED Flex	China

*N* of patients; *n* of small aneurysms (≤10 mm); NR: not report; DAPT: Dual Antiplatelet Therapy; FRED X: Flow-Redirection Endoluminal Device X; PED: Pipeline embolization device.

### 3.3 Quality assessment

Based on the Joanna Briggs Institute (JBI) Critical Appraisal Checklist for Case Series, 10 clinical studies were evaluated across ten domains assessing aspects such as case selection, disease evaluation, and data reporting. The results of quality assessment are provided in Table 2.

**Table 2:** The results of JBI critical appraisal checklist for case series.

Query	Akgul et al. (15)	Brasiliense et al. (16)	Chalouhi et al. (17)	Clausen et al. (18)	Griessenauer et al. (19)	Lin et al. (21)	Kallmes et al. (20)	Roy et al. (22)	Trivelato et al. (23)	Zhang et al. (3)
1	Yes	Yes	Yes	Yes	Yes	Yes	Yes	Yes	Yes	Yes
2	Yes	Yes	Yes	Yes	Yes	Yes	Yes	Yes	Yes	Yes
3	Yes	Yes	Yes	Yes	Yes	Yes	Yes	Yes	Yes	Yes
4	Unclear	Unclear	Yes	Yes	Yes	Yes	Yes	Yes	Yes	Yes
5	Unclear	Unclear	Yes	Yes	Yes	Yes	Yes	Yes	Yes	Yes
6	Yes	Yes	Yes	Yes	Yes	Yes	Yes	Yes	Yes	Yes
7	Yes	Yes	Yes	Yes	Yes	Yes	Yes	Yes	Yes	Yes
8	Yes	Yes	Yes	Yes	Yes	Yes	Yes	Yes	Yes	Yes
9	Yes	Yes	Yes	Yes	Yes	Yes	Yes	Yes	Yes	Yes
10	Yes	Yes	Yes	Yes	Yes	Yes	Yes	Yes	Yes	Yes
Overall appraisal	Include	Include	Include	Include	Include	Include	Include	Include	Include	Include

Query 1: Were there clear criteria for inclusion in the case series? Query 2: Was the condition measured in a standard, reliable way for all participants included in the case series? Query 3: Were valid methods used for identification of the condition for all participants included in the case series? Query 4: Did the case series have consecutive inclusion of participants? Query 5: Did the case series have complete inclusion of participants? Query 6: Was there clear reporting of the demographics of the participants in the study? Query 7: Was there clear reporting of clinical information of the participants? Query 8: Were the outcomes or follow up results of cases clearly reported? Query 9: Was there clear reporting of the presenting site(s)/clinic(s) demographic information? Query 10: Was statistical analysis appropriate?

### 3.4 Meta-analysis results

#### 3.4.1 Complete or near-complete occlusion

**Figure 2:**
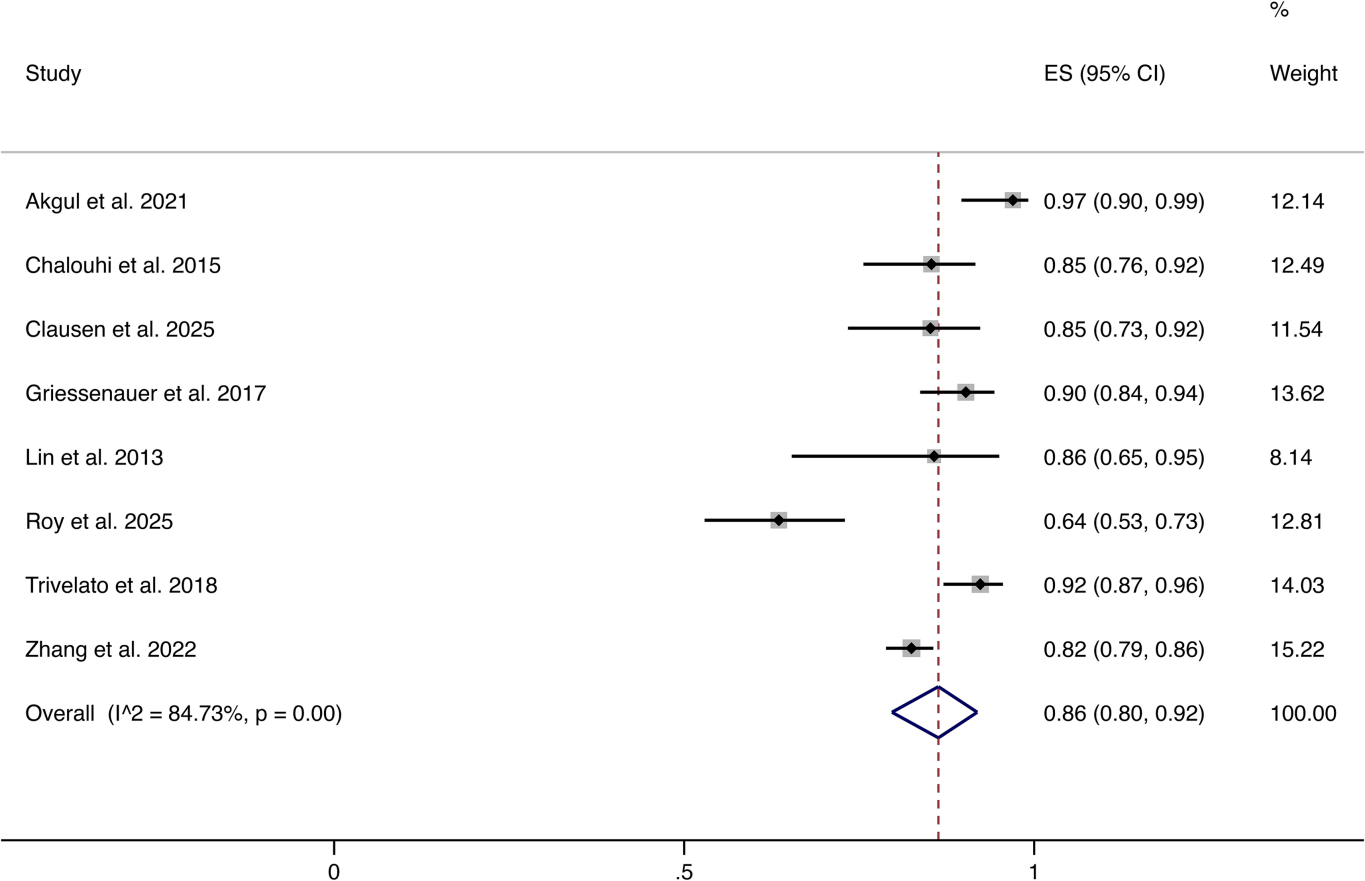
Forest plot of complete or near-complete occlusion.

Eight studies reported complete or near-complete occlusion (Raymond–Roy Grade I). The pooled analysis demonstrated an overall rate of 86% (95% CI: 80–92%). Significant heterogeneity was observed (I^2^ = 84.73%, *p* = 0.00). These findings indicate that most patients achieved favorable angiographic outcomes following FD treatment (shown in Figure 2).

#### 3.4.2 Incomplete occlusion (<90%)

**Figure 3:**
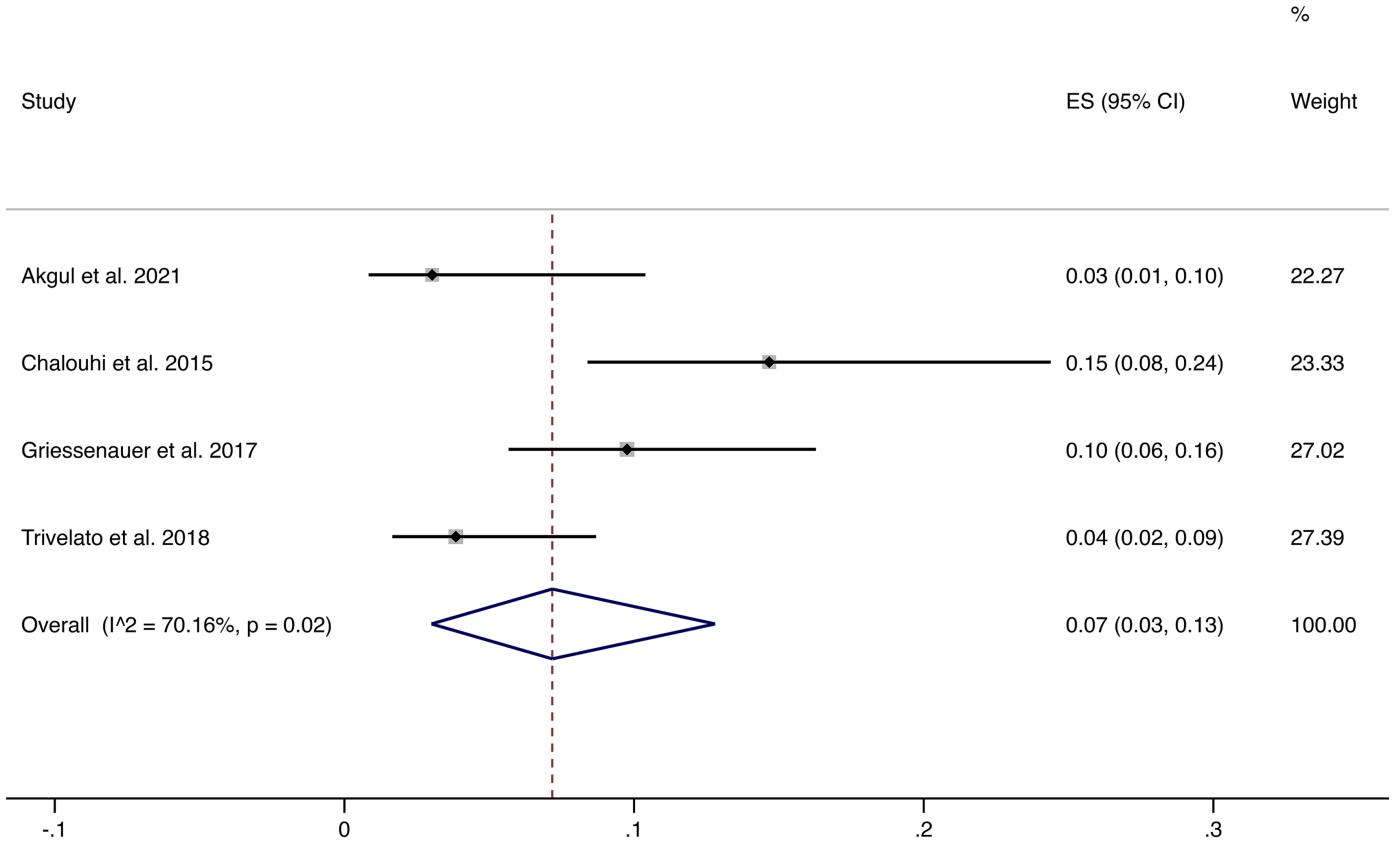
Forest plot of incomplete occlusion (<90%).

Four studies reported incomplete occlusion (<90%). The pooled incidence was 7% (95% CI: 3–13%) with high heterogeneity (I^2^ = 70.16%, *p* = 0.02). This suggests that although most patients achieve complete or near-complete occlusion, a minority may experience incomplete occlusion, warranting careful clinical follow-up (shown in Figure 3).

#### 3.4.3 Mortality

**Figure 4:**
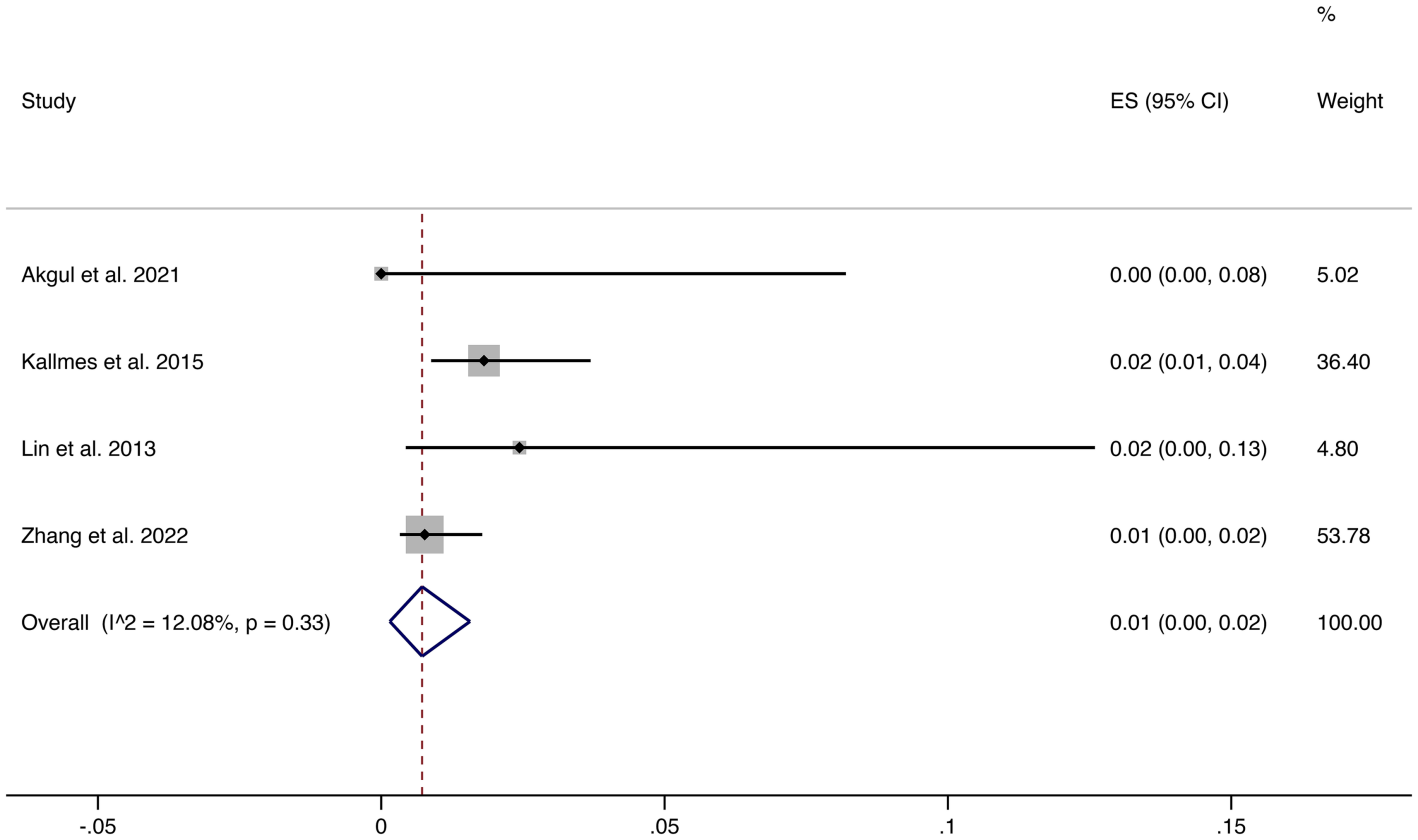
Forest plot of mortality.

Four studies reported mortality outcomes. The pooled mortality rate was 1% (95% CI: 0–2%) with low heterogeneity (I^2^ = 12.08%, *p* = 0.33), suggesting robust and consistent results. This indicates that FD treatment is associated with a very low risk of treatment-related death (shown in Figure 4).

#### 3.4.4 Hemorrhagic events

**Figure 5:**
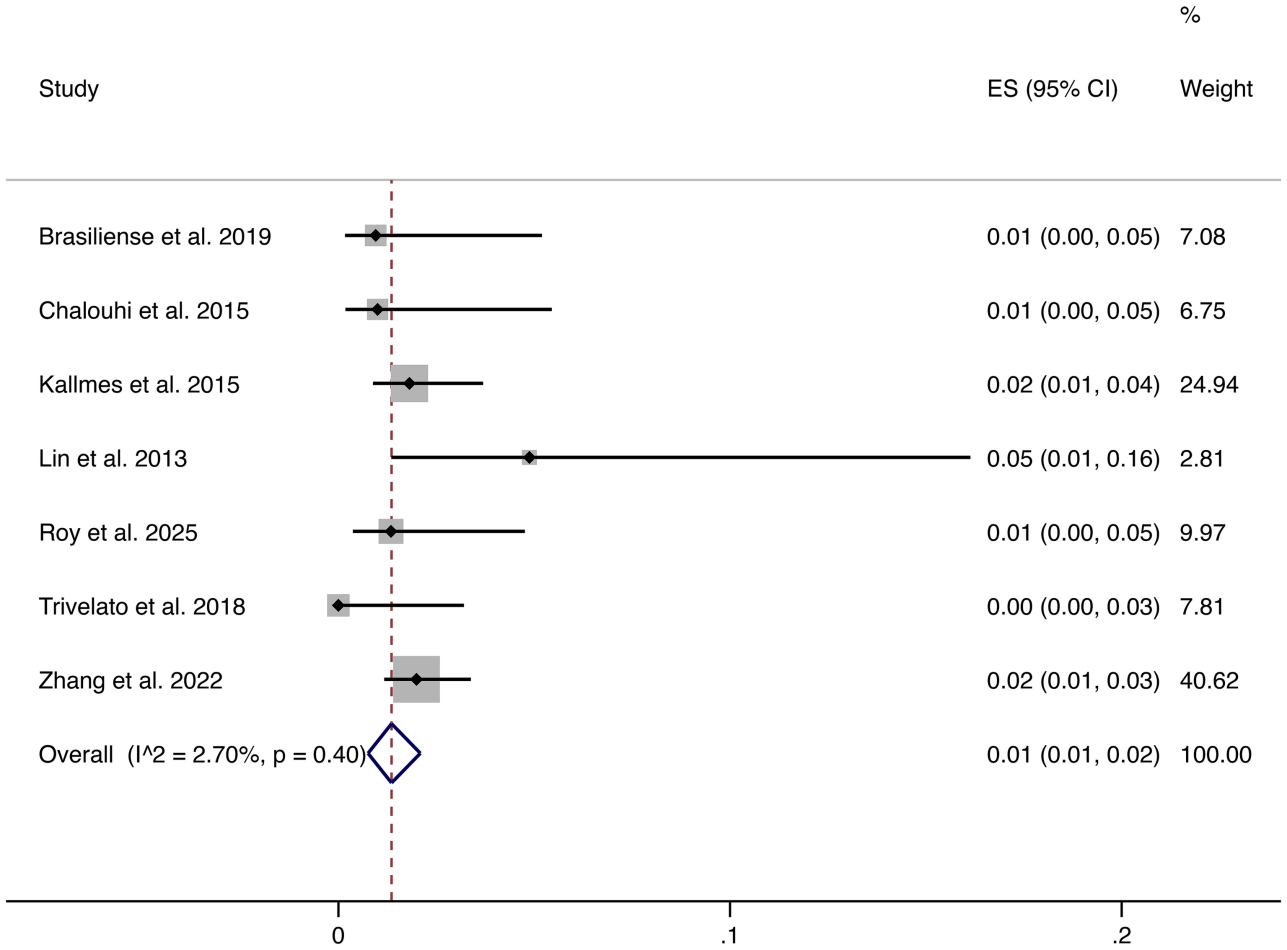
Forest plot of hemorrhagic events.

Seven studies reported hemorrhagic complications. The pooled rate was 1% (95% CI: 1–2%), with very low heterogeneity (I^2^ = 2.70%, *p* = 0.40), indicating highly consistent findings across studies. These results confirm that hemorrhagic risk following FD treatment is low (shown in Figure 5).

#### 3.4.5 Stroke

**Figure 6:**
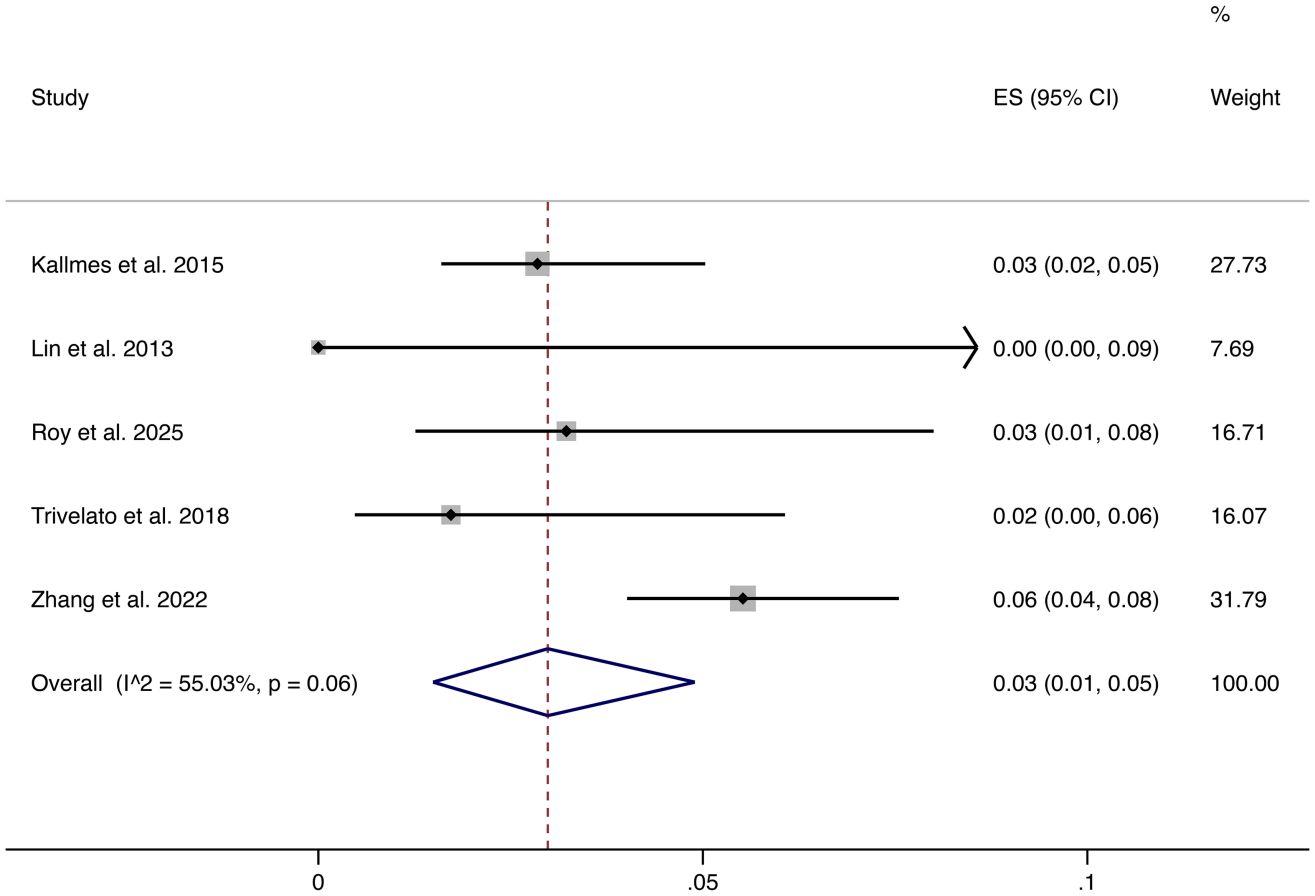
Forest plot of stroke.

Five studies reported stroke as an outcome. The pooled rate was 3% (95% CI: 1–5%) with moderate heterogeneity (I^2^ = 55.03%, *p* = 0.06). Although relatively uncommon, stroke remains a clinically relevant complication, underscoring the importance of perioperative risk management (shown in Figure 6).

#### 3.4.6 Ischemic events

**Figure 7:**
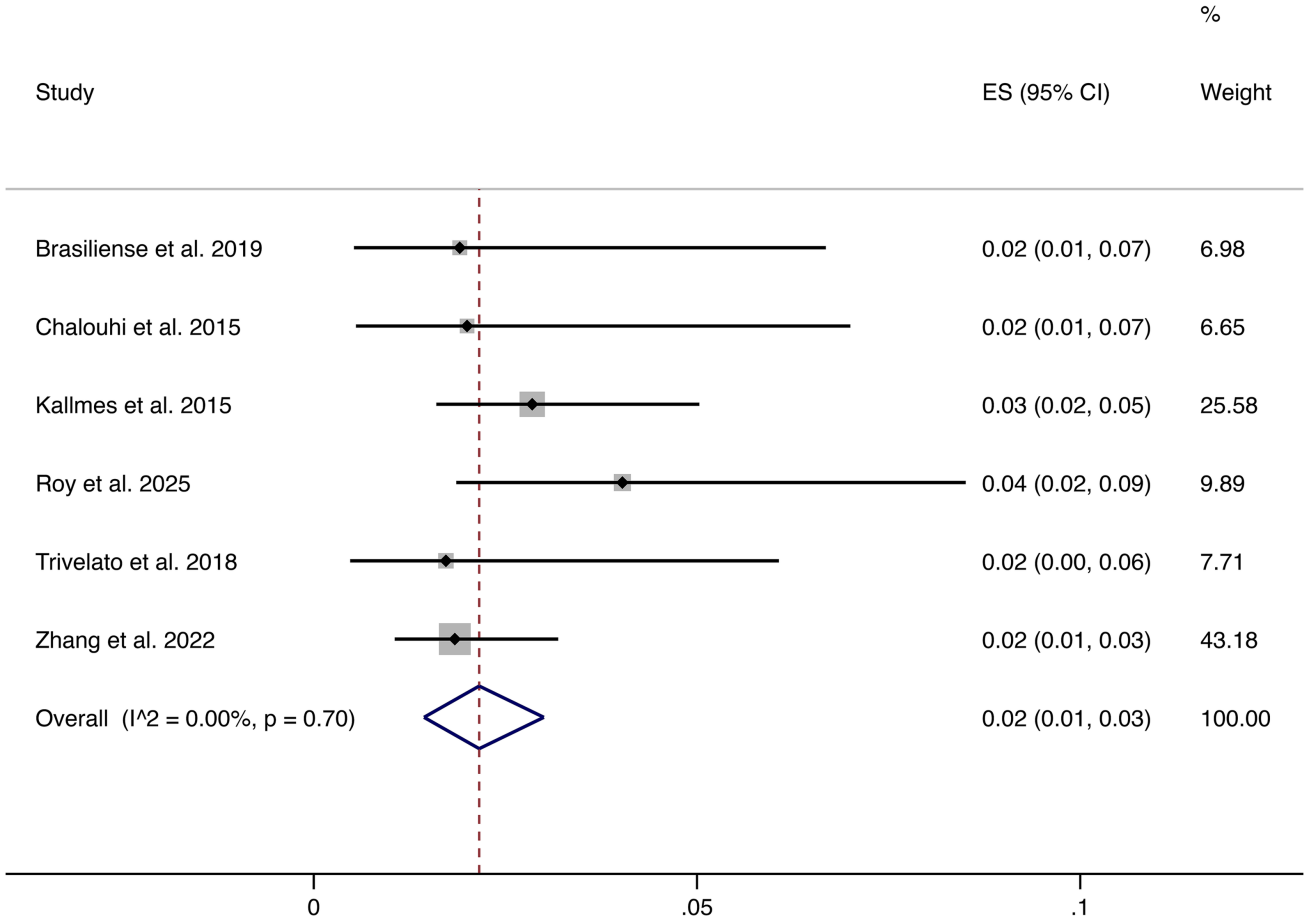
Forest plot of ischemic events.

Six studies reported ischemic complications. The pooled rate was 2% (95% CI: 1–3%), with low heterogeneity (I^2^ = 0.00%, *p* = 0.70). This demonstrates that ischemic risk is consistently low across studies (shown in Figure 7).

#### 3.4.7 Favorable functional outcomes

**Figure 8:**
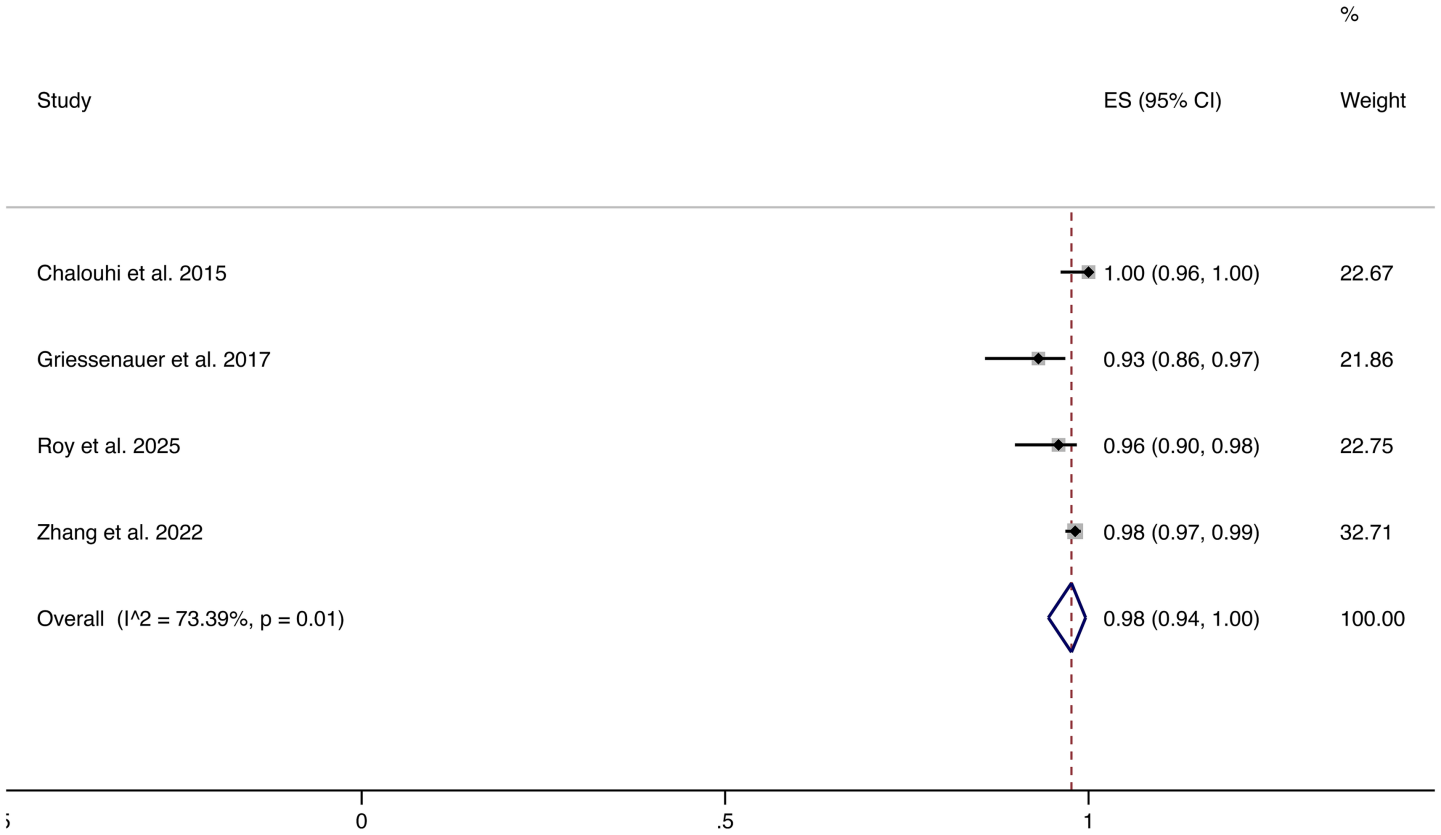
Forest plot of favorable functional outcomes.

Four studies reported favorable functional outcomes (mRS 0–2). The pooled incidence was 98% (95% CI: 94–100%), although substantial heterogeneity was observed (I^2^ = 73.39%, *p* = 0.01). These results suggest that most patients maintained good neurological function following FD treatment (shown in Figure 8).

#### 3.4.8 Complications

**Figure 9:**
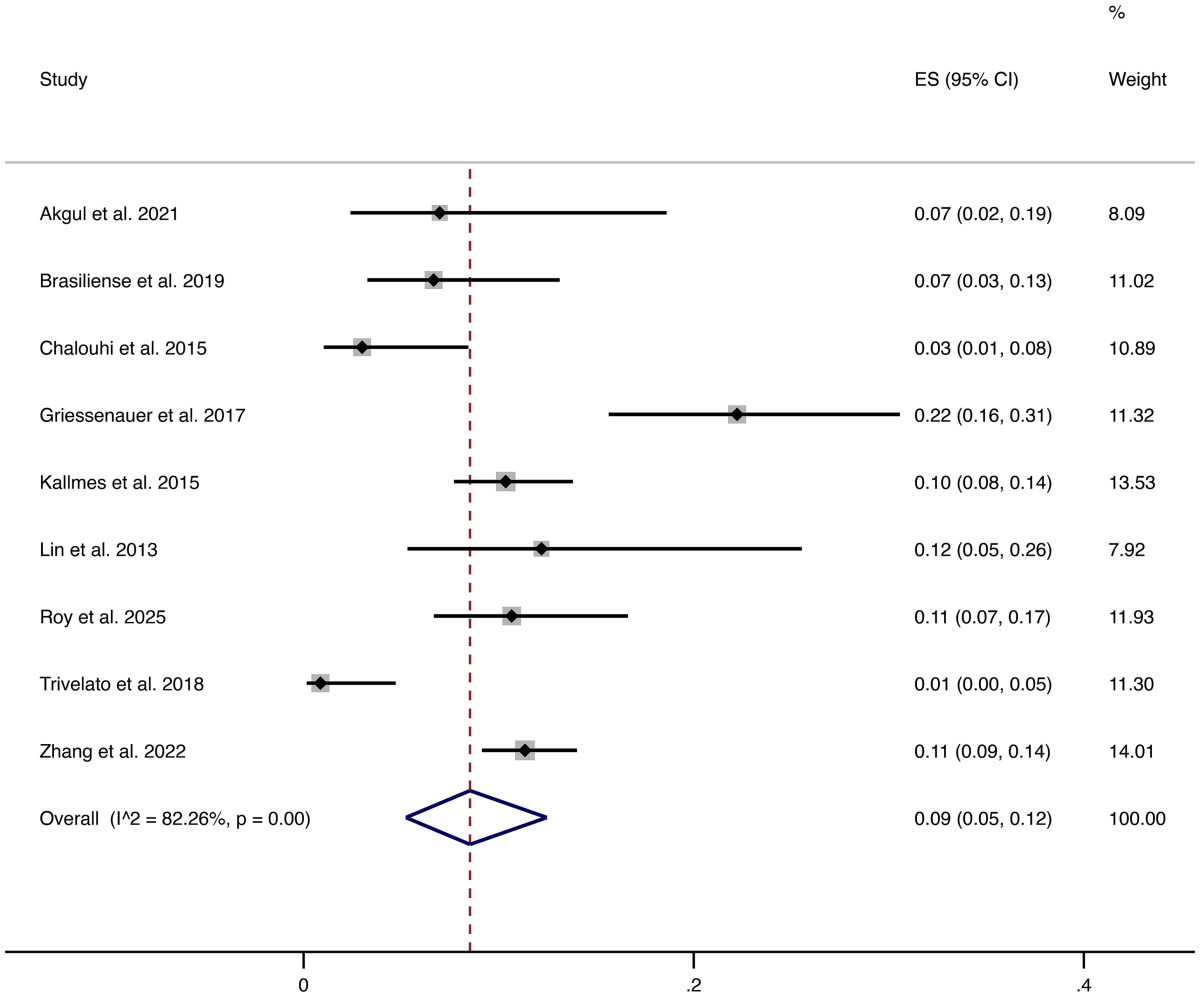
Forest plot of overall complications.

Nine studies reported overall complications. The pooled complication rate was 9% (95% CI: 5–12%), with high heterogeneity (I^2^ = 82.26%, *p* = 0.00). This indicates considerable variability in reported complication rates across studies, highlighting that although FD treatment is generally safe, certain patients remain at risk of peri- or post-procedural complications that require clinical vigilance (shown in Figure 9).

### 3.5 Publication bias

The funnel plot suggested some asymmetry, indicating the potential presence of publication bias or small-study effects. However, Egger’s regression test yielded *p* = 0.791, suggesting no statistically significant evidence of publication bias; thus, the results should be interpreted with caution.

## 4 Discussion

This meta-analysis included 10 retrospective studies comprising 2,275 patients with 1,938 SIAs. The pooled analysis demonstrated a complete or near-complete occlusion rate of 86%, indicating high efficacy of FDs in the treatment of small aneurysms. In addition, the rates of treatment-related mortality and hemorrhage were both 1%, ischemic events occurred in 2%, stroke in 3%, and favorable functional outcomes were achieved in 98% of patients. The overall complication rate was 9%. These findings suggest that FDs provide not only excellent angiographic outcomes but also favorable safety and functional profiles in the management of small intracranial aneurysms.

SIAs present unique therapeutic challenges due to their small size and complex anatomy. Conventional treatment approaches, such as microsurgical clipping or endovascular coiling, often face limitations including incomplete occlusion or recurrence. By reconstructing parent vessel hemodynamics, FDs promote intra-aneurysmal thrombosis and vessel wall remodeling, thereby achieving progressive occlusion. The present study indicates that in SIAs, FDs can achieve high occlusion rates without substantially increasing the risk of severe complications, which holds important clinical implications.

Our findings are largely consistent with previous prospective studies and meta-analyses, though several methodological differences should be considered. The PREMIER trial investigated unruptured wide-neck aneurysms ≤12 mm and reported complete occlusion rates of 76.8% at one year and 83.3% at three years, with a primary safety event rate of 2.8% (11, 24). However, the PREMIER cohort included both small (<10 mm) and medium-sized (10–12 mm) aneurysms, whereas our meta-analysis focused only on small aneurysms. Differences in aneurysm size, baseline characteristics, and patient selection likely account for part of the discrepancy. Bhatia et al. reported a major complication rate of only 0.9% in <10 mm aneurysms treated with the Pipeline Flex device, which is much lower than our pooled rate (25). This is largely due to methodological differences. Bhatia defined complications strictly as major adverse events (death, major ischemic stroke, or symptomatic intracranial hemorrhage), while our analysis included all reported complications, both major and minor. Their review also focused only on the second-generation Pipeline Flex, whereas our study covered multiple flow diverters across different devices and settings. Fiorella et al. conducted a meta-analysis of predominantly small to medium sized unruptured ICA aneurysms (26). They reported a 12-month complete occlusion rate of 74.9% and a composite primary safety event rate of 7.8%, providing performance benchmarks that are broadly in line with our findings. In our pooled analysis, the complete or near-complete occlusion rate was 86%, slightly higher than some prospective studies, while the overall complication rate was 9%. This higher rate likely reflects the greater heterogeneity of retrospective studies, variation in device type and operator experience, and differences in antiplatelet regimens. Nevertheless, when broken down by event type, treatment-related mortality and hemorrhagic events were 1%, ischemic events 2%, and stroke 3%. These remain lower than rates reported in cohorts of large or giant aneurysms. Finally, the proportion of patients with favorable functional outcomes reached 98%, reinforcing the clinical benefit of flow diverter treatment in small aneurysms.

This study has several limitations. First, all included studies were retrospective in design, which may introduce selection bias. Second, the relatively small sample sizes of some studies limit the robustness of the pooled estimates. Third, variability in follow-up duration and imaging assessment criteria across studies may have contributed to heterogeneity in the results. Fourth, most of the included studies mixed ruptured and unruptured small aneurysms, with only a few reporting results separately. Because of limited available data, we were unable to conduct a subgroup meta-analysis by rupture status. Future studies should stratify and report outcomes by rupture status to reduce heterogeneity and improve the quality of evidence. Therefore, future large-scale, multicenter, prospective randomized controlled trials with standardized imaging assessments and long-term follow-up are warranted to further validate the efficacy and safety of FDs in SIAs.

## 5 Conclusion

In this systematic review and single-arm meta-analysis, flow diverters demonstrated high efficacy and acceptable safety in the treatment of small intracranial aneurysms. With an overall complete occlusion rate of 86% and a favorable functional outcome rate of 98%, FD treatment offers a promising therapeutic option. Nonetheless, the high heterogeneity and retrospective design of included studies highlight the need for prospective, multicenter randomized trials to validate these findings and establish standardized clinical protocols.

## Data Availability

The original contributions presented in the study are included in the article/Supplementary material, further inquiries can be directed to the corresponding authors.
