# Supplementary material for: Efficacy and safety of flow diverters in small intracranial aneurysms: a systematic review and single-arm meta-analysis
Source: Front Neurol. 2025 Nov 26;16:1706462. doi: 10.3389/fneur.2025.1706462 (PMC12689322; doi:10.3389/fneur.2025.1706462)

**Supplementary Materials**

**Supplementary file 1:** Search strategy (PubMed)

| No. | Query | Results |
| --- | --- | --- |
| #1 | "Intracranial Aneurysm"[MeSH Terms] | 33,772 |
| #2 | "intracranial aneurysm"[Title/Abstract] OR "cerebral aneurysm"[Title/Abstract] OR "brain aneurysm"[Title/Abstract] OR "small intracranial aneurysms"[Title/Abstract] | 13,057 |
| #3 | #1 OR #2 | 37,612 |
| #4 | "flow diverter"[Title/Abstract] OR "flow diversion"[Title/Abstract] OR "flow diverting stent"[Title/Abstract] OR "pipeline embolization device"[Title/Abstract] OR "fred stent"[Title/Abstract] OR "p64 flow modulation device"[Title/Abstract] OR "woven endobridge device"[Title/Abstract] OR "web device"[Title/Abstract] OR "endovascular stent"[Title/Abstract] OR "intracranial stent"[Title/Abstract] | 6,725 |
| #5 | #3 AND #4 | 2,667 |

**Supplementary file 2:** Funnel plot and results of egger’s test


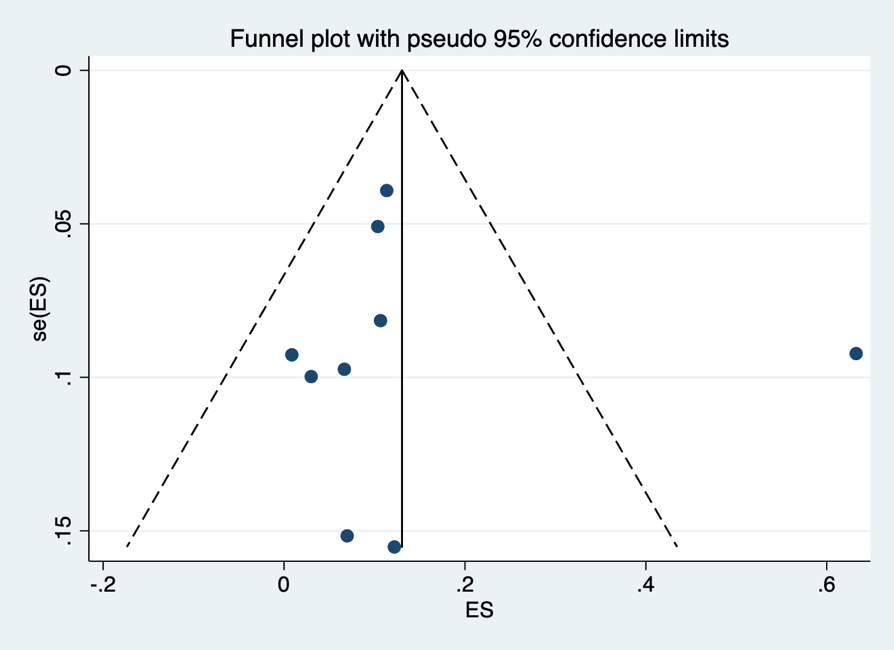


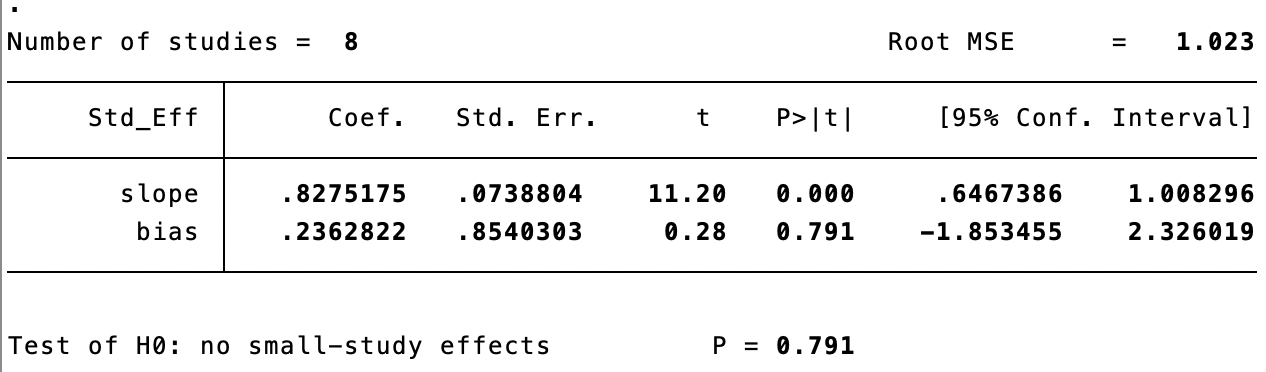

Supplement: Supplementary file 1 [file Table_1.docx]
